# Supplementary material for: Anti Gram-Positive Bacteria Activity of Synthetic Quaternary Ammonium Lipid and Its Precursor Phosphonium Salt
Source: Int J Mol Sci. 2024 Feb 27;25(5):2761. doi: 10.3390/ijms25052761 (PMC10931589; doi:10.3390/ijms25052761)
Supplement: Supplementary file 1 [file ijms-25-02761-s001.zip › ijms-2878814-supplementary.pdf]

International Journal of *Molecular Sciences*

MDPI

Supplementary Materials

Anti Gram-Positive Bacteria Activity of Synthetic Quaternary Lipid and its Precursor Phosphonium Salt

Francesca Bacchetti [1,†], Anna Maria Schito [2,†], Marco Milanese [1,3], Sara Castellaro [1], and Silvana Alfei [1,*]

[1] Department of Pharmacy, University of Genoa, Viale Cembrano, 16148 Genoa, Italy; francesca.bacchetti@edu.unige.it (F.B.); sara.caste@libero.it (S.C.), marco.milanese@ unige.it (M.M.)

[2] Department of Surgical Sciences and Integrated Diagnostics (DISC), University of Genoa, Viale Benedetto XV, 6, I-16132 Genova, Italy; amschito@unige.it (A.M.S)

[3] IRCCS Ospedale Policlinico San Martino, Genoa, Italy

†These authors contributed equally

* Correspondence: alfei@difar.unige.it; Tel.: +39 010 355 2296 (S.A.)

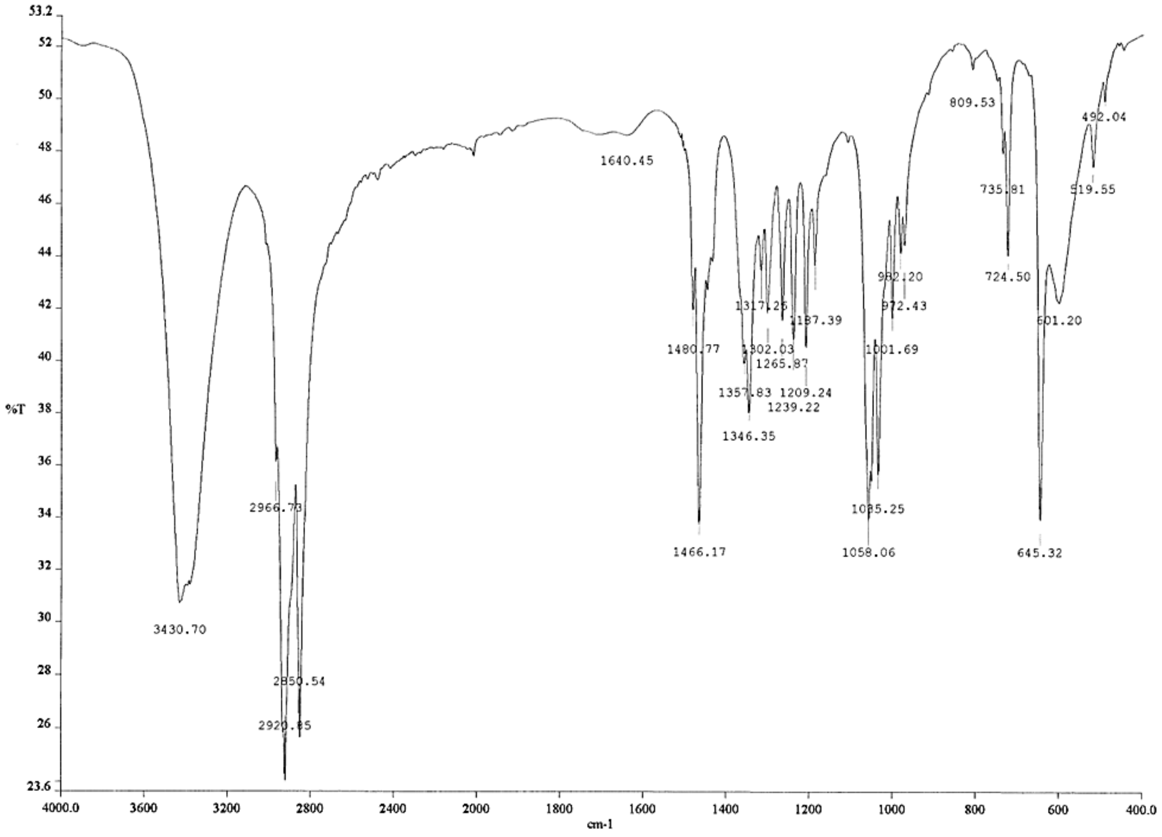

Figure S1. FTIR spectrum of 11-bromo-undecan-1-ol (oil on KBr disc).

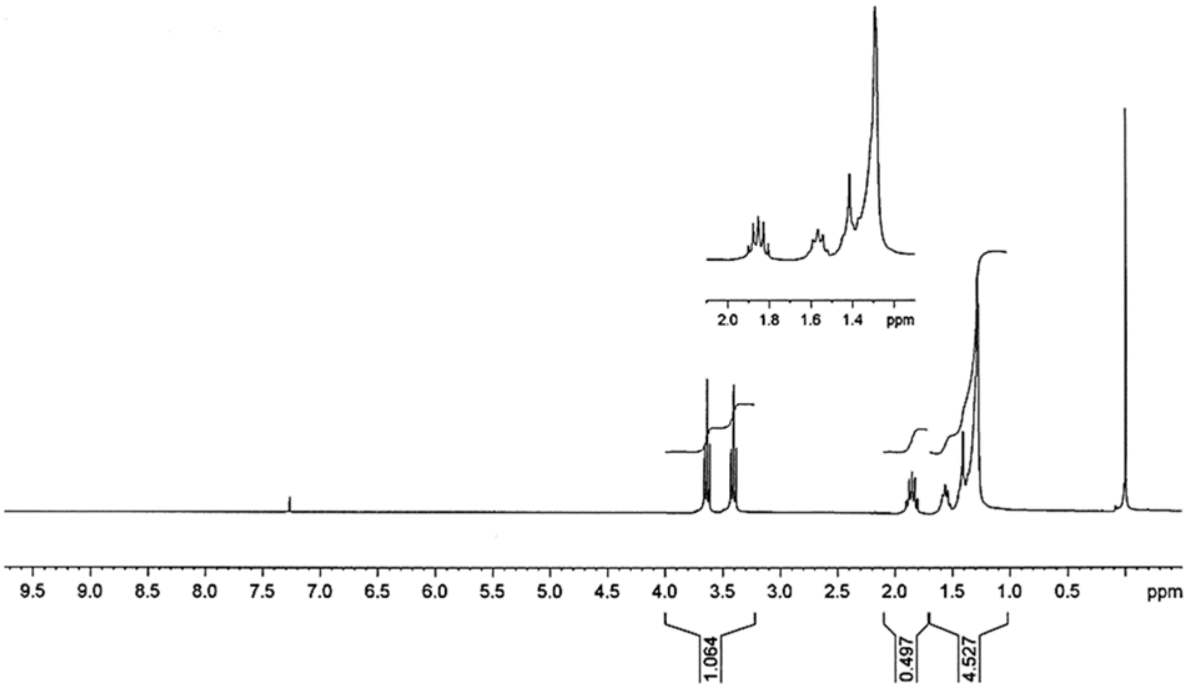

Figure S2. ^{1}H NMR spectrum ($CDCl_3$, 300 MHz) spectrum of 11-bromo-undecan-1-ol.

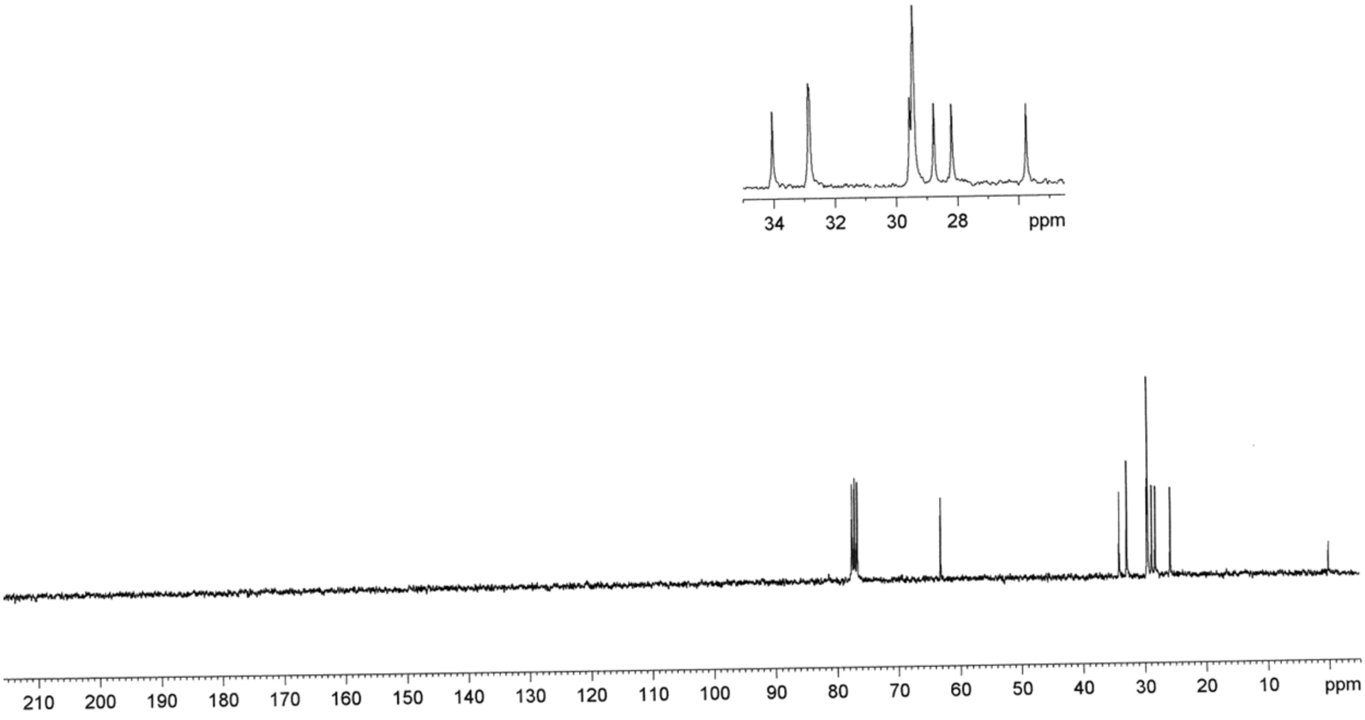

Figure S3. ^{13}C NMR spectrum ($CDCl_3$, 75.5 MHz) spectrum of 11-bromo-undecan-1-ol.

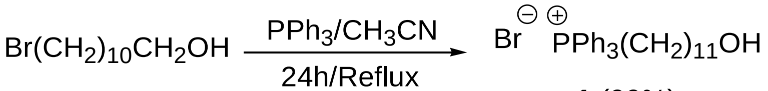

1 (99%)

Scheme S1. Synthesis of (11-hydroxyundecyl) triphenyl phosphonium bromide (**1**).

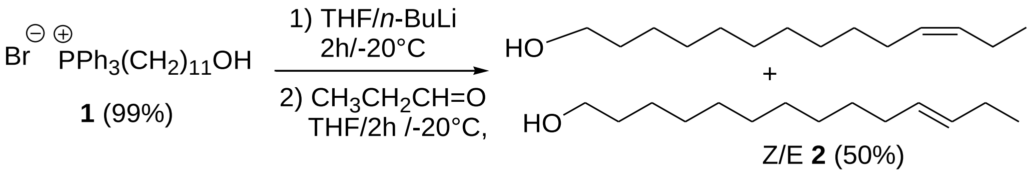

Scheme S2. Synthesis of the (*Z*/*E*)-11-tetradecen-1-ol (**2**).

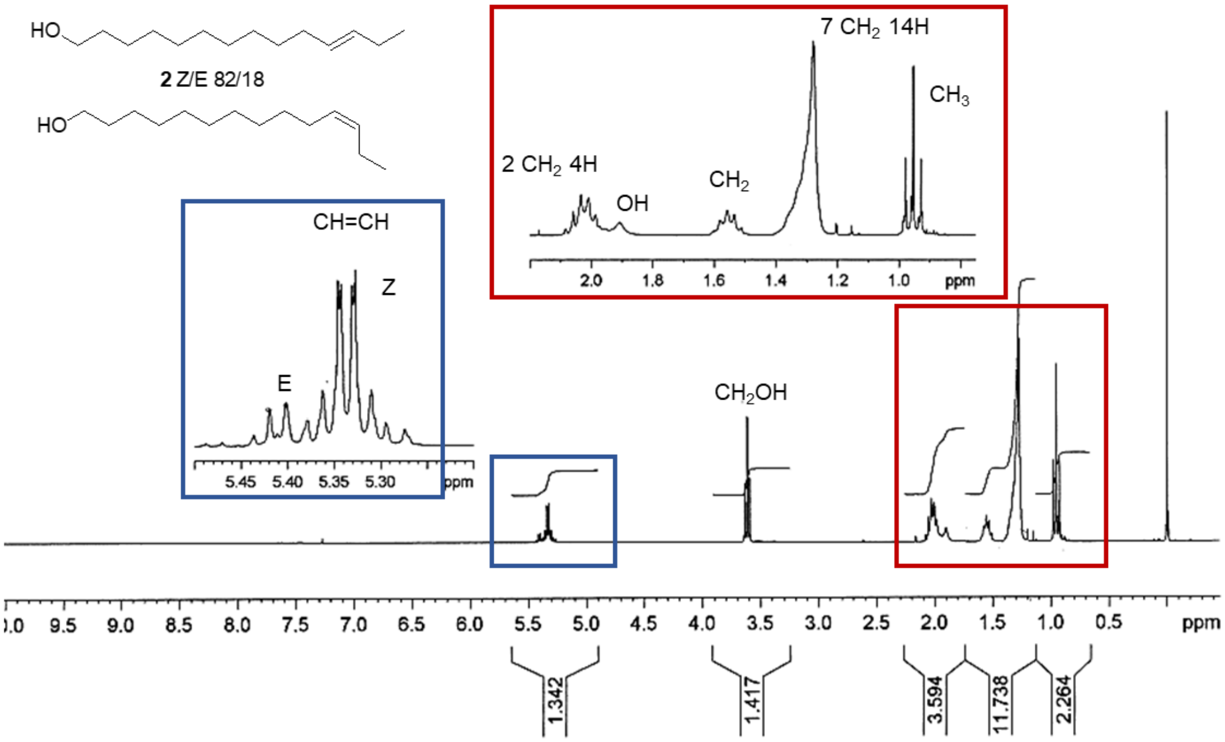

Figure S4. ^{1}H NMR spectrum (CDCl3, 300 MHz) spectrum of Z/E-11-tetradecen-1-ol (**2**). In the squares of the same color are evidenced significant selected regions of the spectrum and the related magnification.

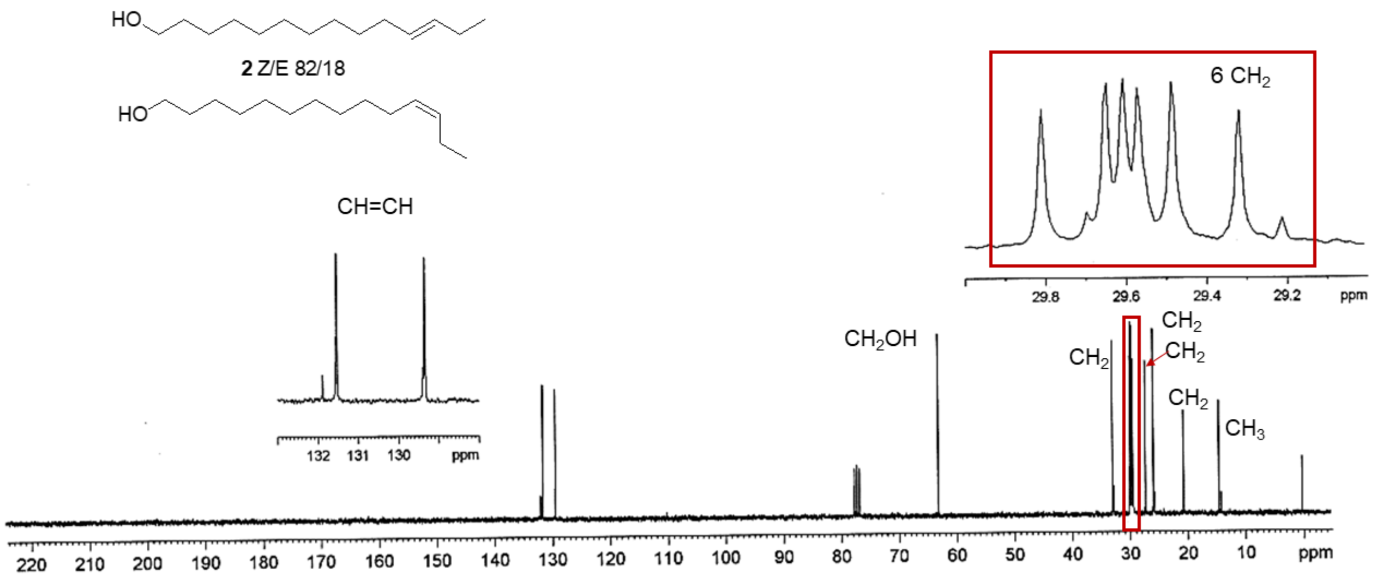

Figure S5. ^{13}C NMR spectrum ($CDCl_3$, 75.5 MHz) spectrum of Z/E-11-tetradecen-1-ol (**2**). In the squares of the same color are evidenced significant selected regions of the spectrum and the related magnification.

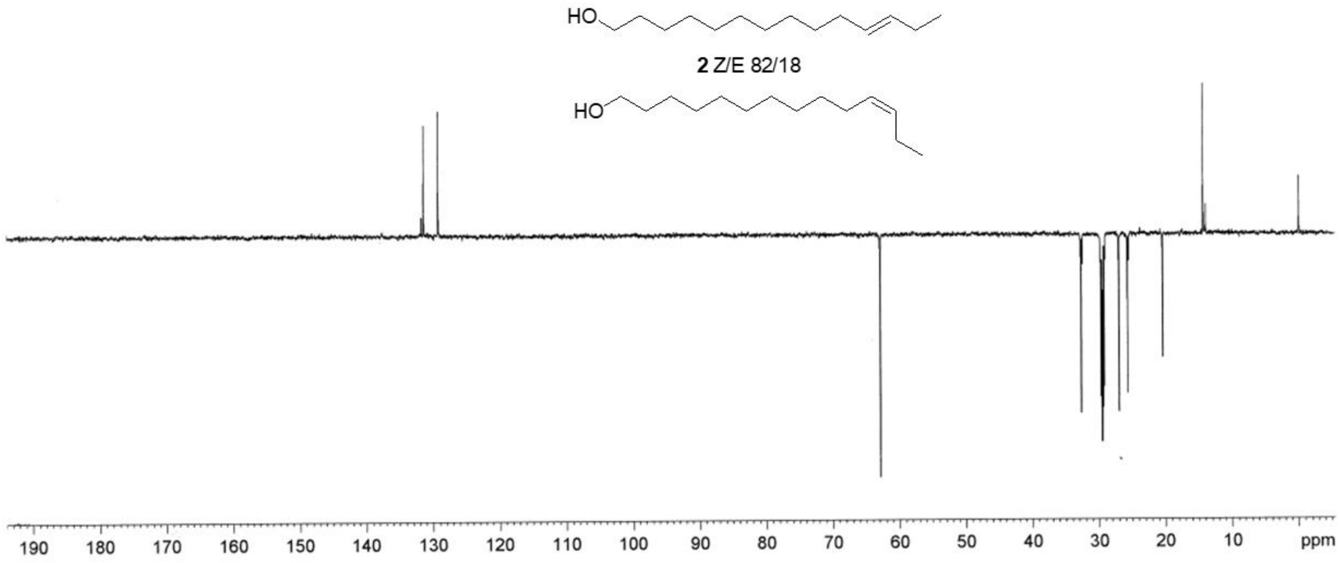

Figure S6. ^{13}C NMR spectrum ($CDCl_3$, 75.5 MHz) spectrum of Z/E-11-tetradecen-1-ol (**2**), 135 DEPT experiment.

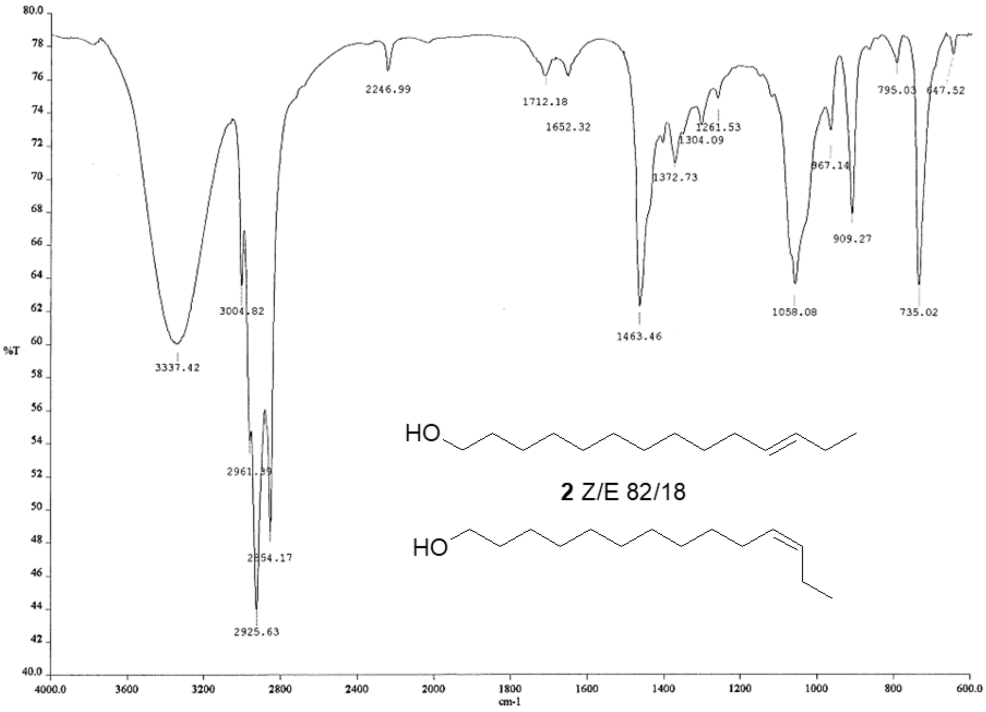

Figure S7. FTIR spectrum (oil on KBr disc) of **2**.

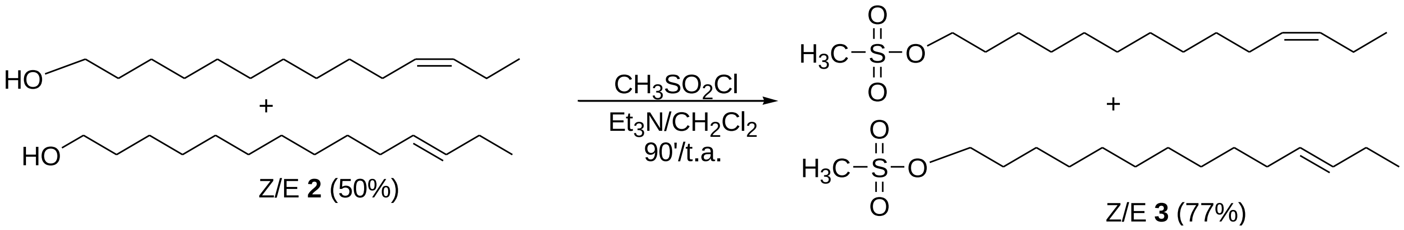

Scheme S3. Synthesis of the (*Z*/*E*)-11-tetradecenyl-1-mesylate (**3**).

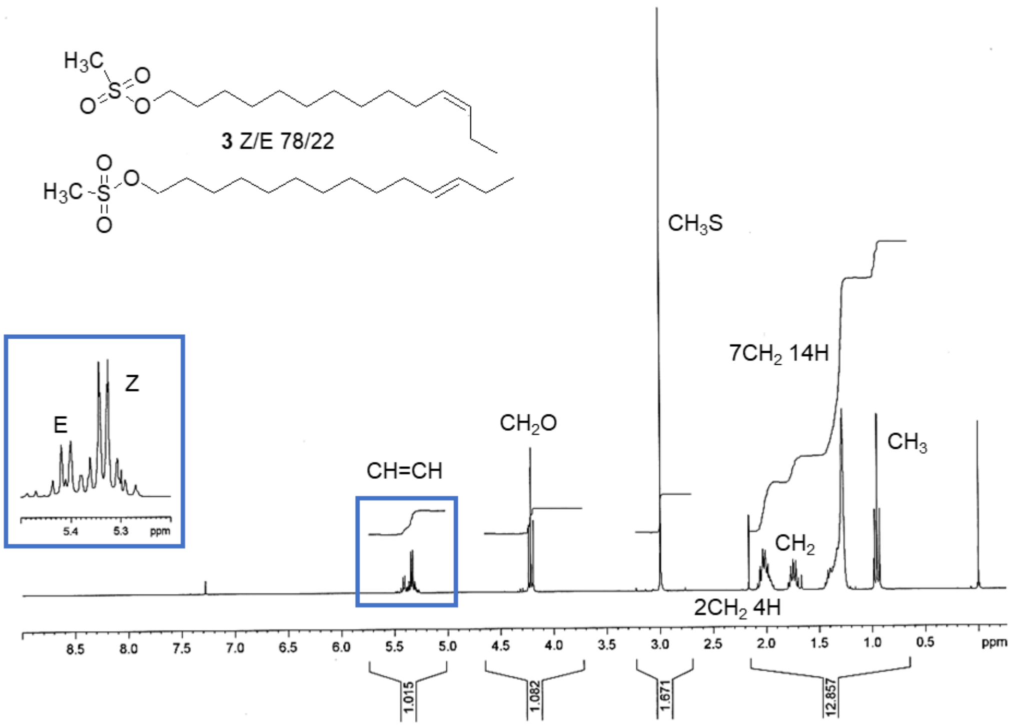

Figure S8. ^{1}H NMR spectrum ($CDCl_3$, 300 MHz) of **3**. In the squares are evidenced significant selected regions of the spectrum and the related magnification.

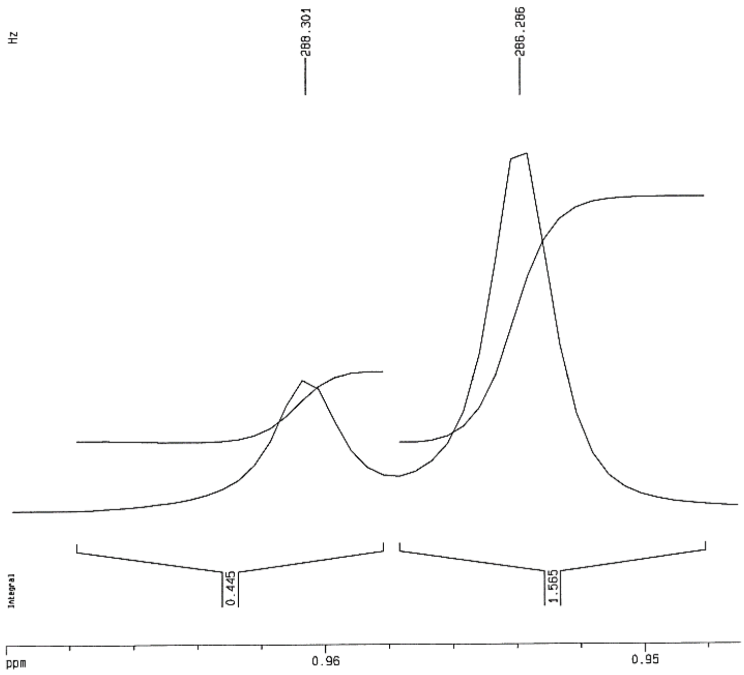

Figure S9. ¹H NMR spectrum ($CDCl_3$, 300 MHz) of **3**: magnification of the signal used to calculate the Z (higher peak)/E (lower peak) ratio.

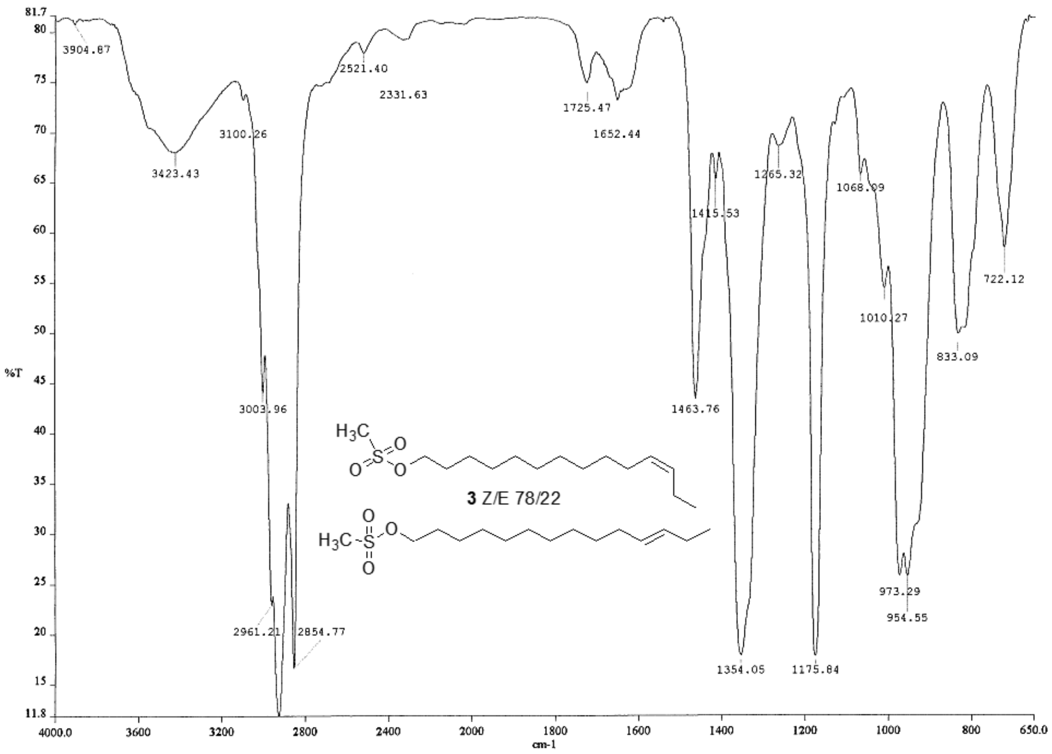

Figure S10. FTIR spectrum (oil on KBr disc) of **3**.

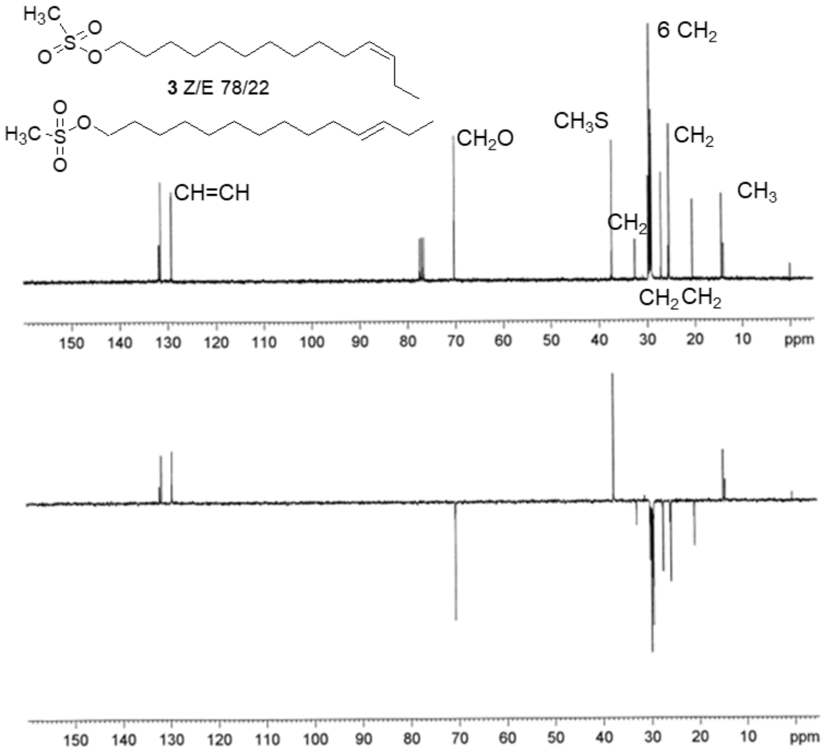

Figure S11. ^{13}C NMR spectrum ($CDCl_3$, 75.5 MHz) of Z/E **3** and DEPT-135 experiment.

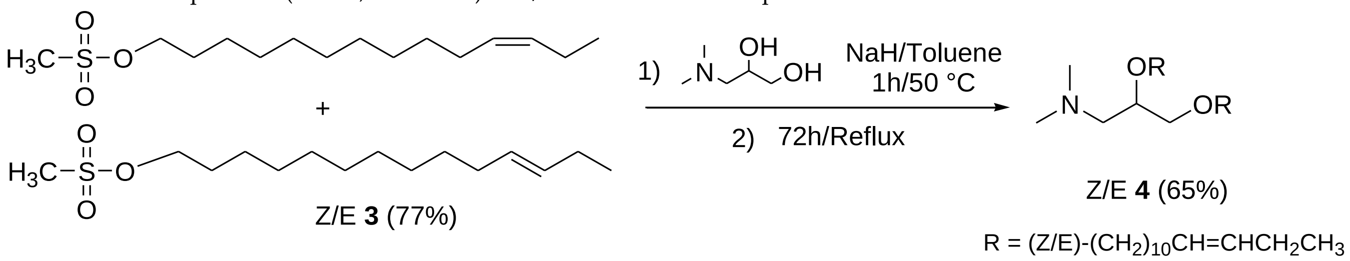

Scheme S4. Synthesis of the *N*,*N*-dimethyl-2,3-bis(tetradec-11-enyloxy)propylamine (**4**).

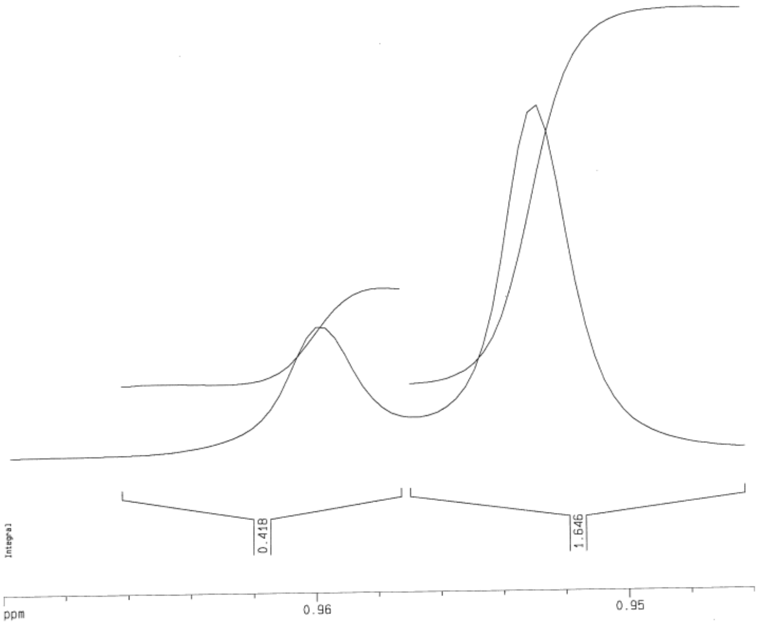

Figure S12. ^{1}H NMR spectrum ($CDCl_3$, 300 MHz) of **4**: magnification of the signal used to calculate the *Z* (higher peak)/*E* (lower peak) ratio.

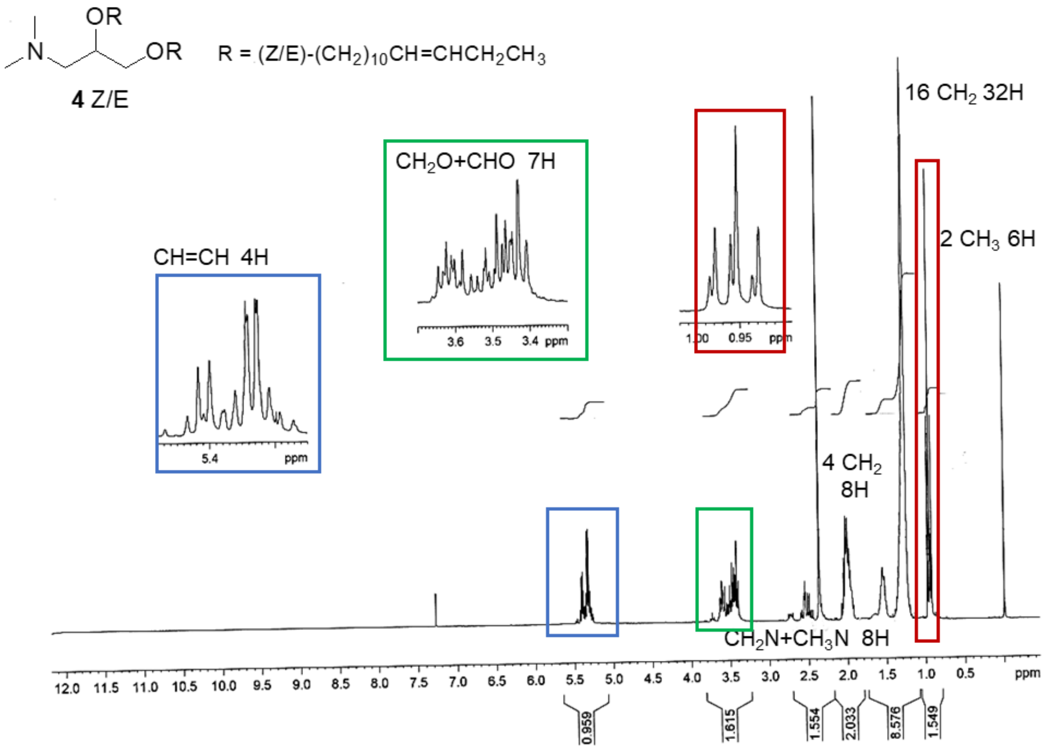

Figure S13. ^{1}H NMR spectrum (CDCl3, 300 MHz) of **4**. In the squares of the same color are evidenced significant selected regions of the spectrum and the related magnification.

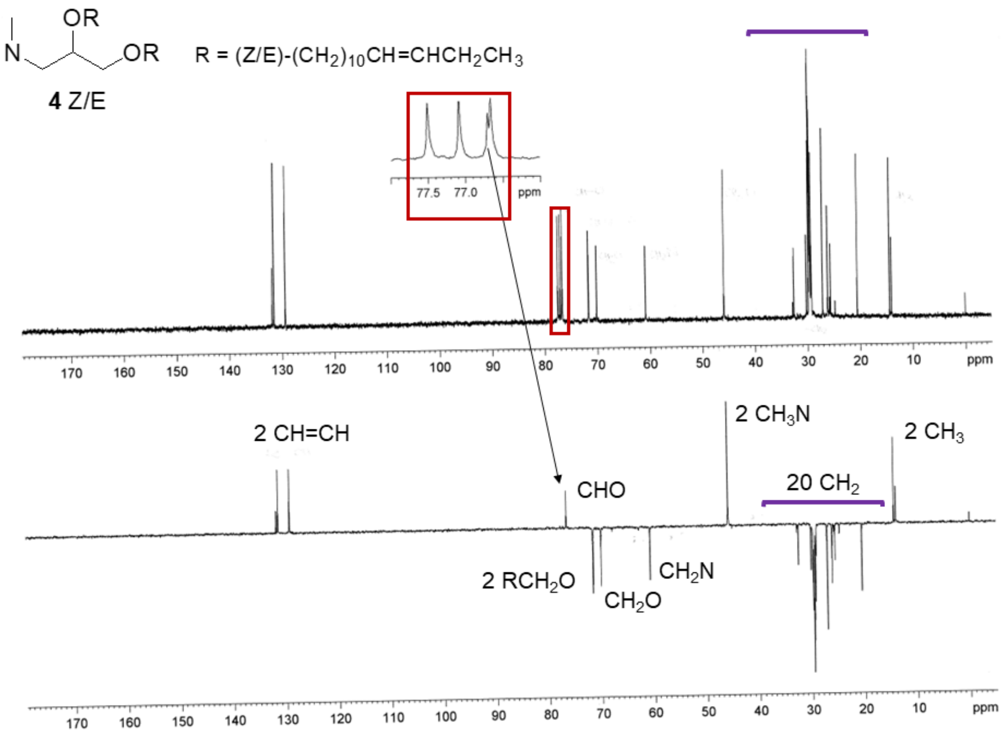

Figure S14. ^{13}C NMR spectrum ($CDCl_3$, 75.5 MHz) of Z/E **4** and DEPT-135 analysis. In the squares are evidenced significant selected regions of the spectrum and the related magnification. The black arrow evidences the CHO peack well visible in the DEPT-135 analysis where chloroform peaks disappeared, while visible only in the magnification in the ^{13}C NMR spectrum. Purple bar groups the signals of the 20 methylene groups.

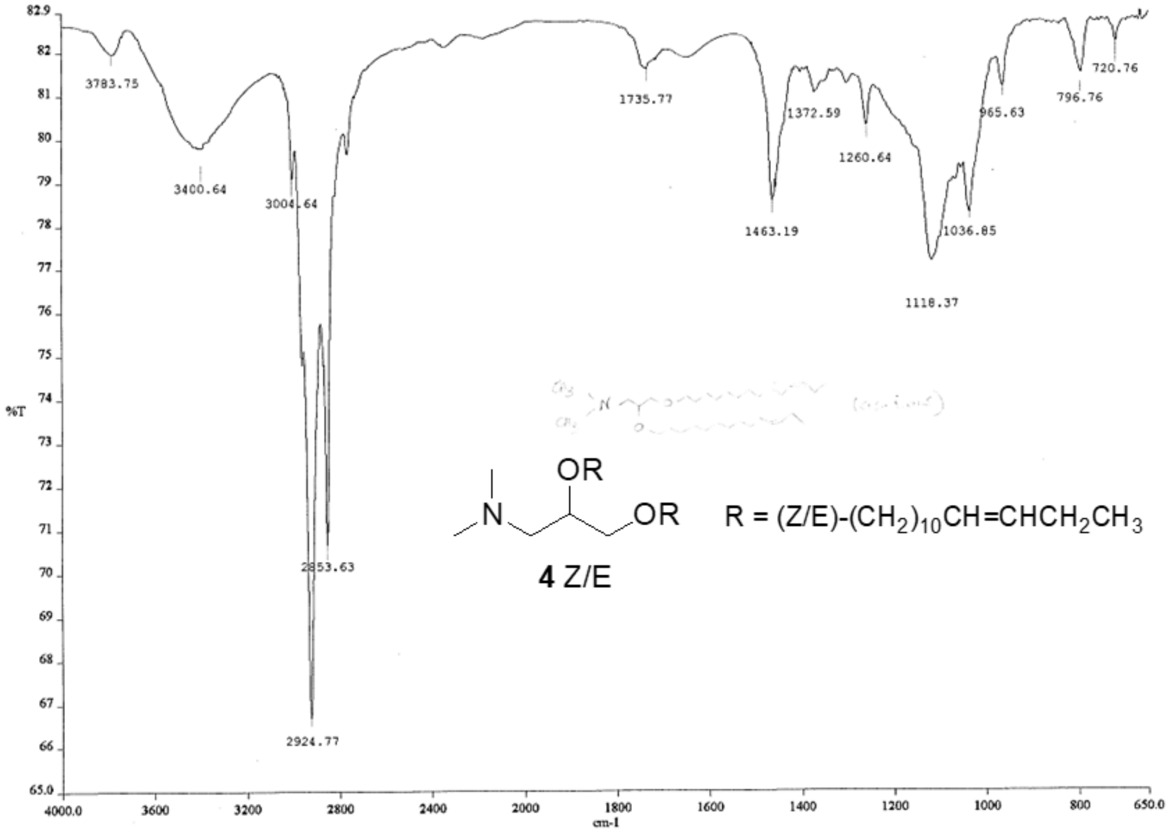

Figure S15. FTIR spectrum (oil on KBr disc) of **4**.

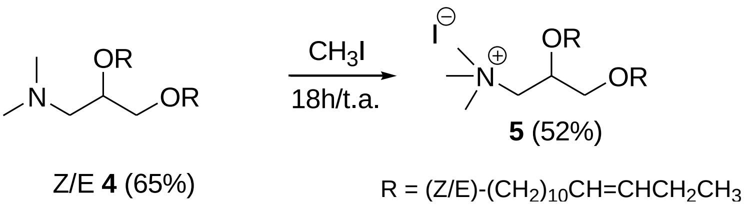

Scheme S5. *N,N,N*-trimethyl-2,3-bis(tetradec-11-enyloxy)propylammonium iodide (**5**).

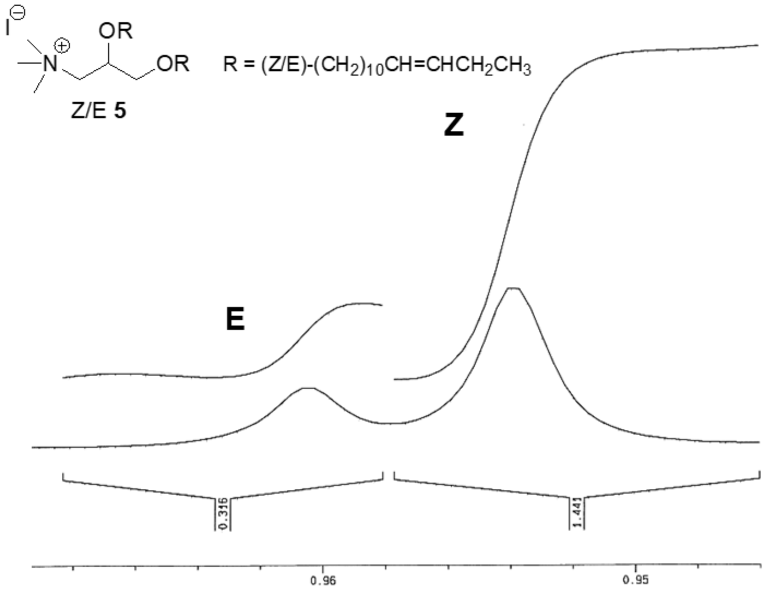

Figure S16. ^{1}H NMR spectrum ($CDCl_3$, 300 MHz) of **4**: magnification of the signal used to calculate the Z/E ratio.

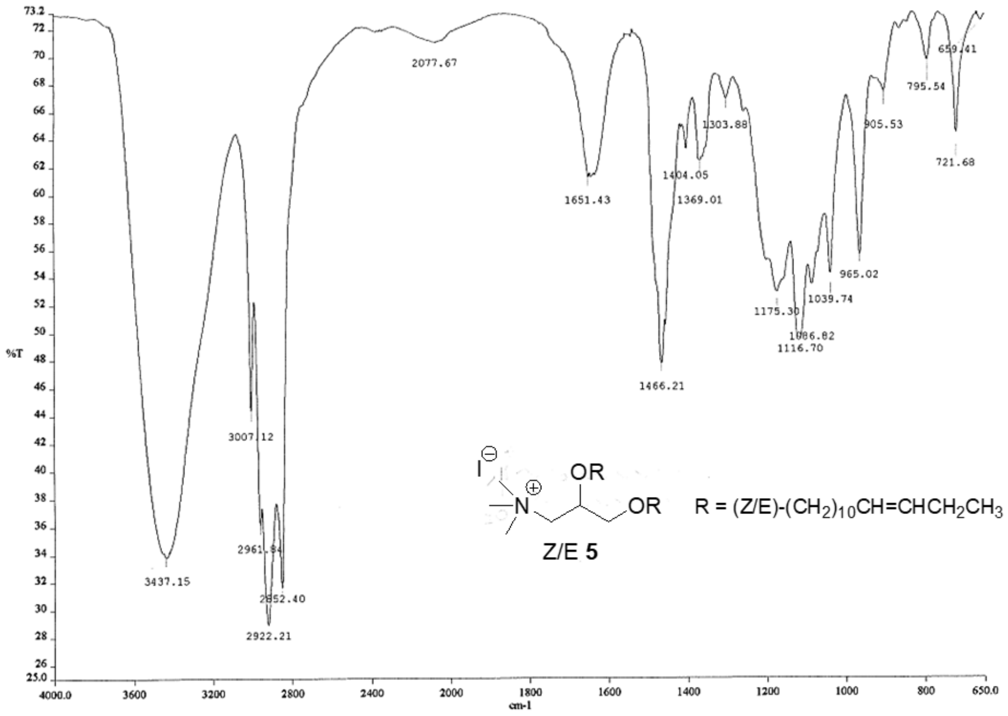

Figure S17. FTIR spectrum (oil on KBr disc) of **5**.

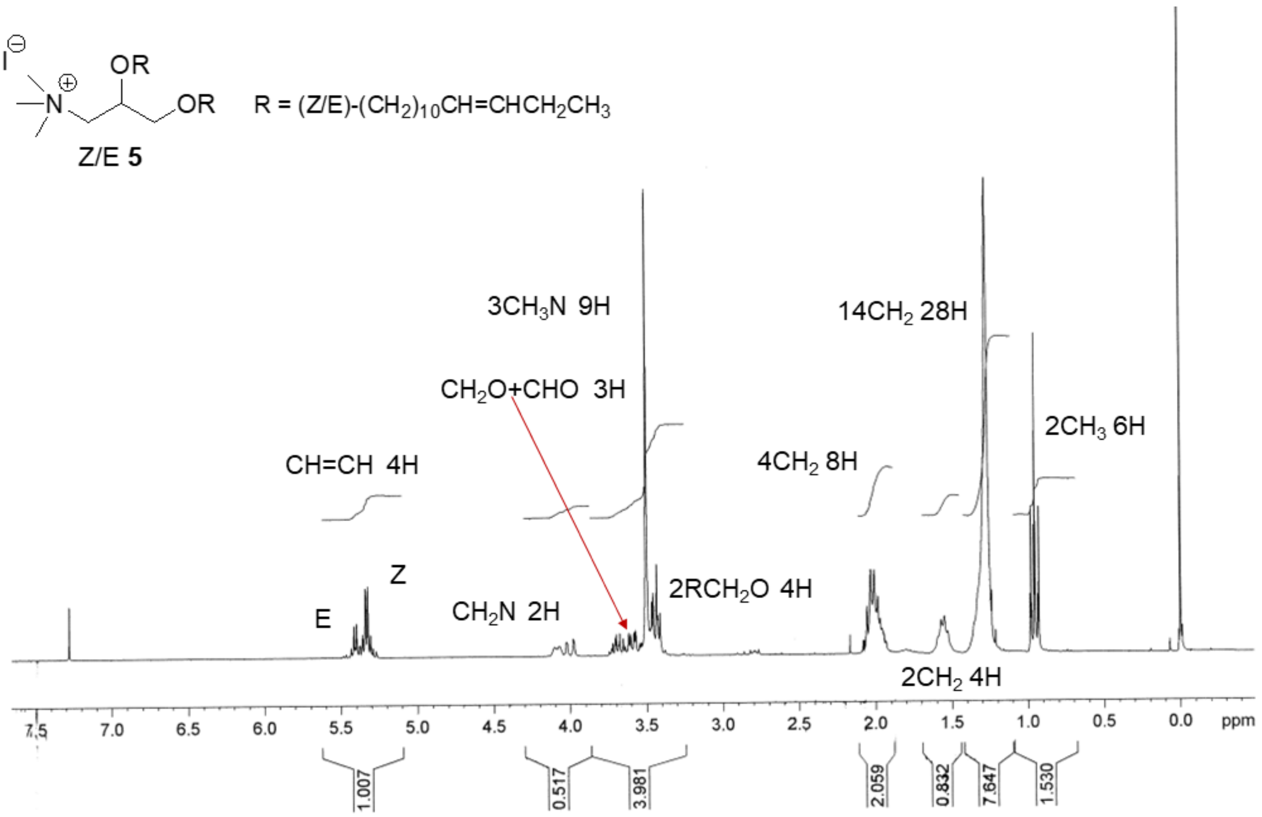

Figure S18. ^{1}H NMR spectrum ($CDCl_3$, 300 MHz) of **4**. The red arrow indicates the signals of CHO and CH_2O proton atoms.

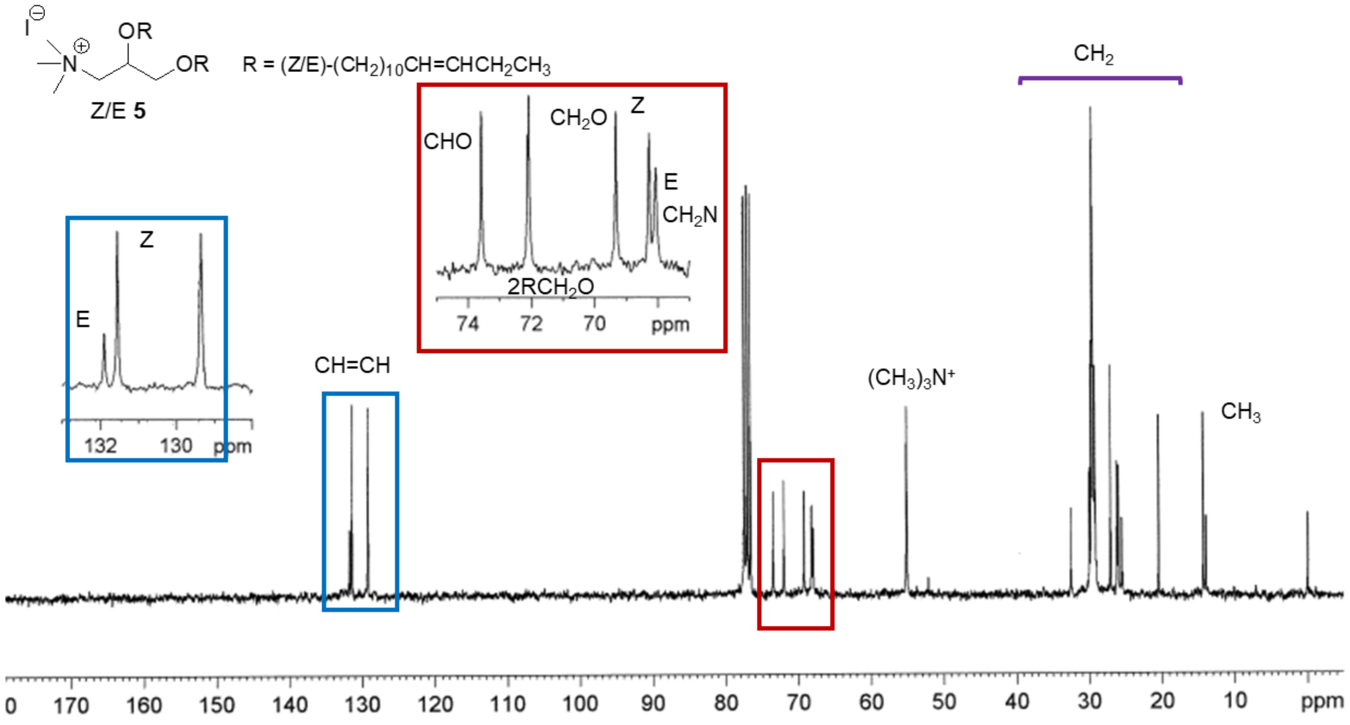

Figure S19. ^{13}C NMR spectrum ($CDCl_3$, 75.5 MHz) of Z/E **5**. In the squares of same color are evidenced significant selected regions of the spectrum and the related magnification. Purple bar groups the signals of the 20 methylene groups.

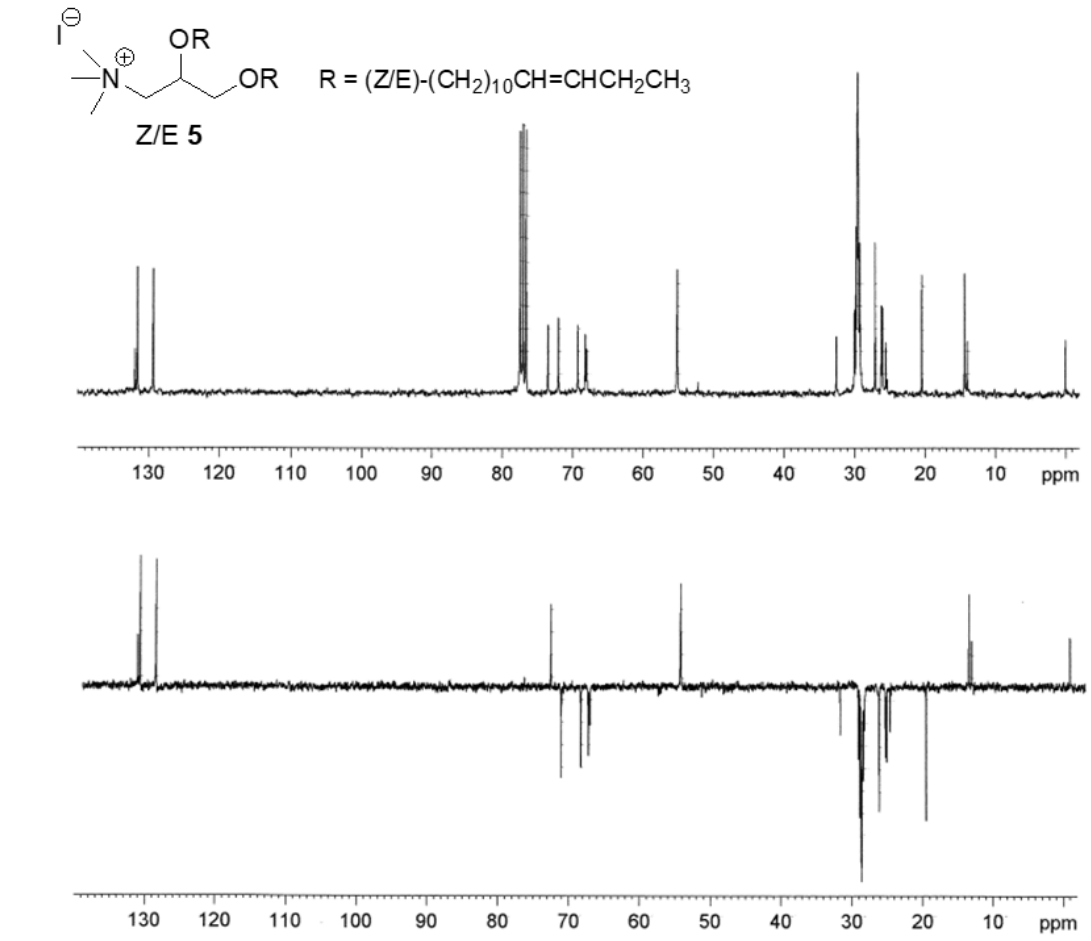

Figure S20. ^{13}C NMR spectrum ($CDCl_3$, 75.5 MHz) of Z/E **5** and its DEPT-135 analysis.

.

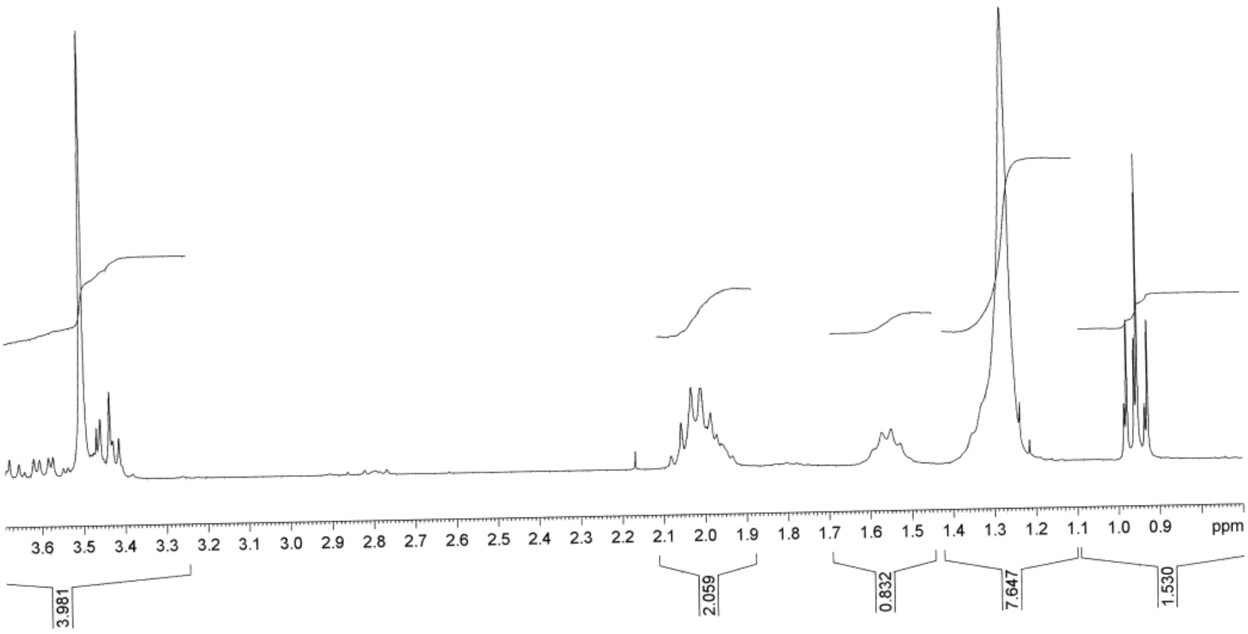

Figure S21. Magnification of the ^{1}H NMR spectrum ($CDCl_3$, 300 MHz) of Z/E **5** in the region 0.8-3.65 ppm.

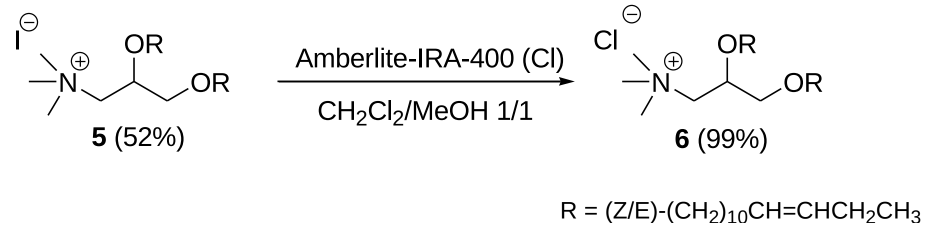

Scheme S6. *N,N,N*-trimethyl-2,3-bis(tetradec-11-enyloxy)propylammonium chloride (**5**).

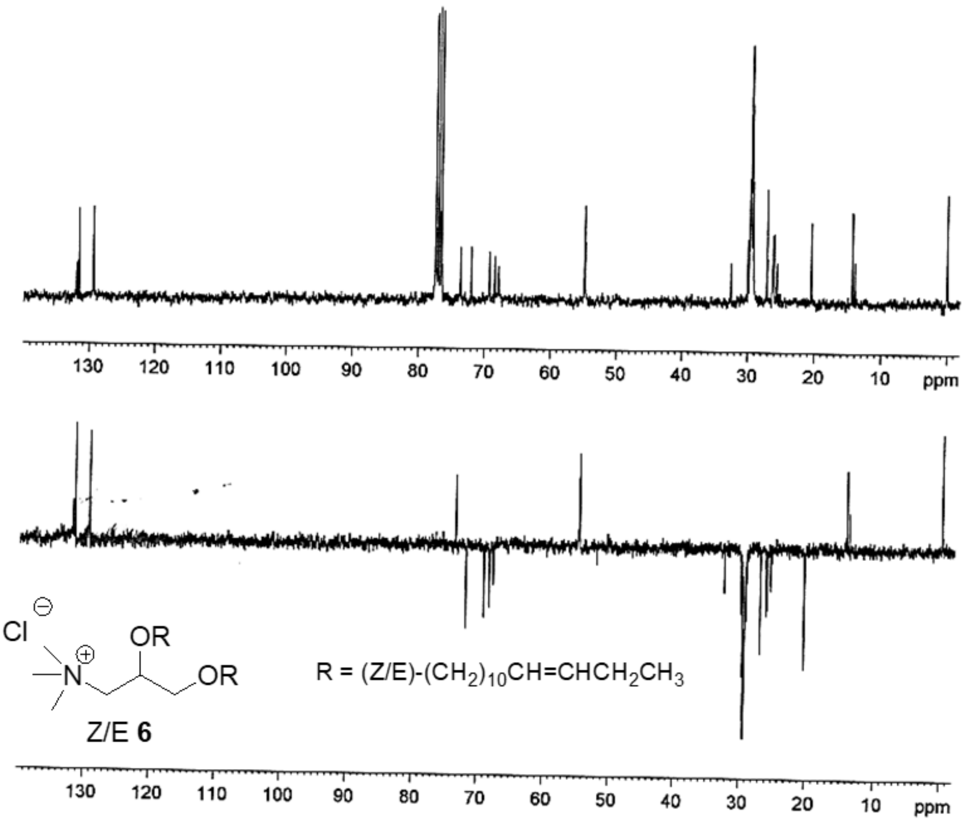

Figure S22. ^{13}C NMR spectrum ($CDCl_3$, 75.5 MHz) of Z/E **6** and its DEPT-135 analysis.

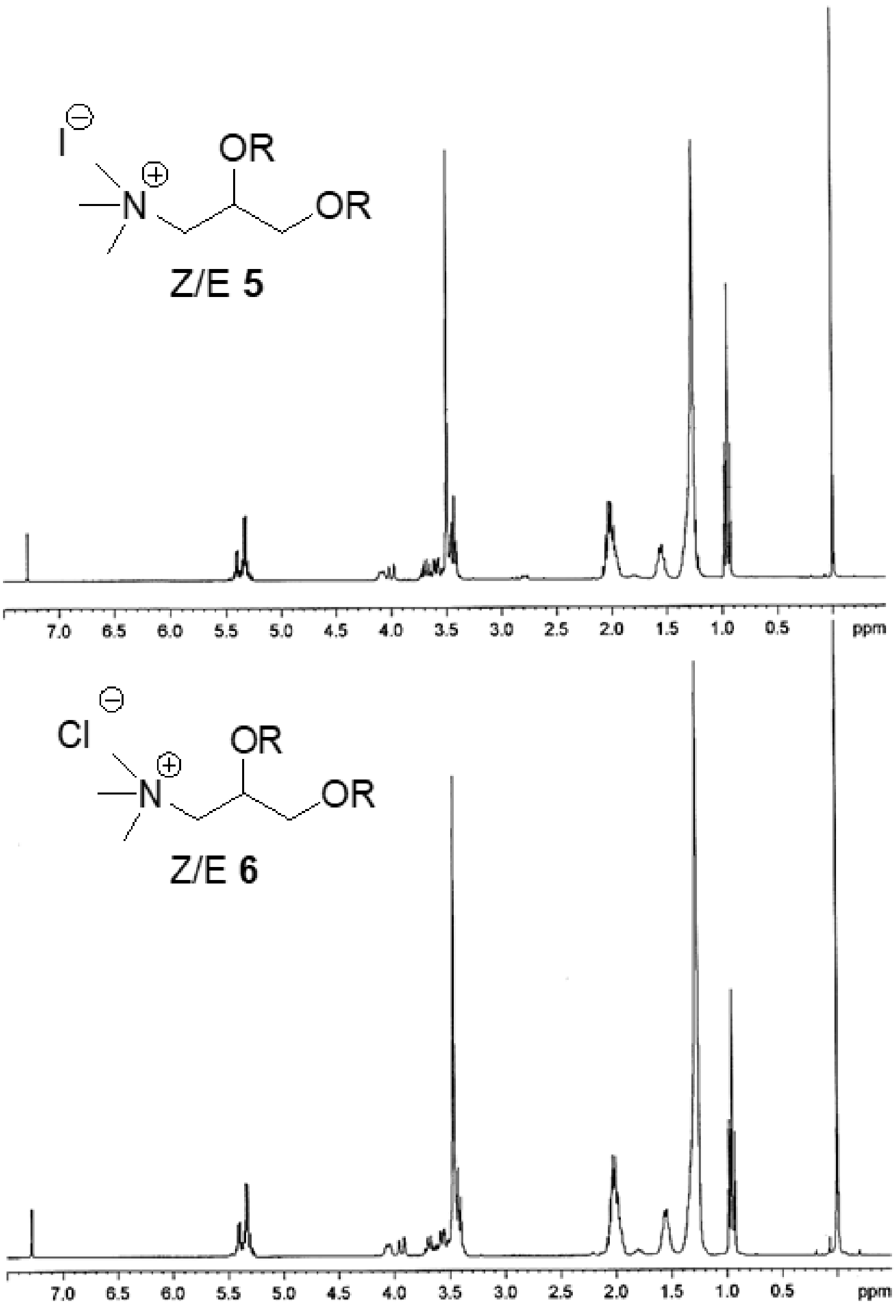

Figure S23. Comparison between the ^{1}H NMR spectra ($CDCl_3$, 300 MHz) of Z/E **5** and Z/E **6**.

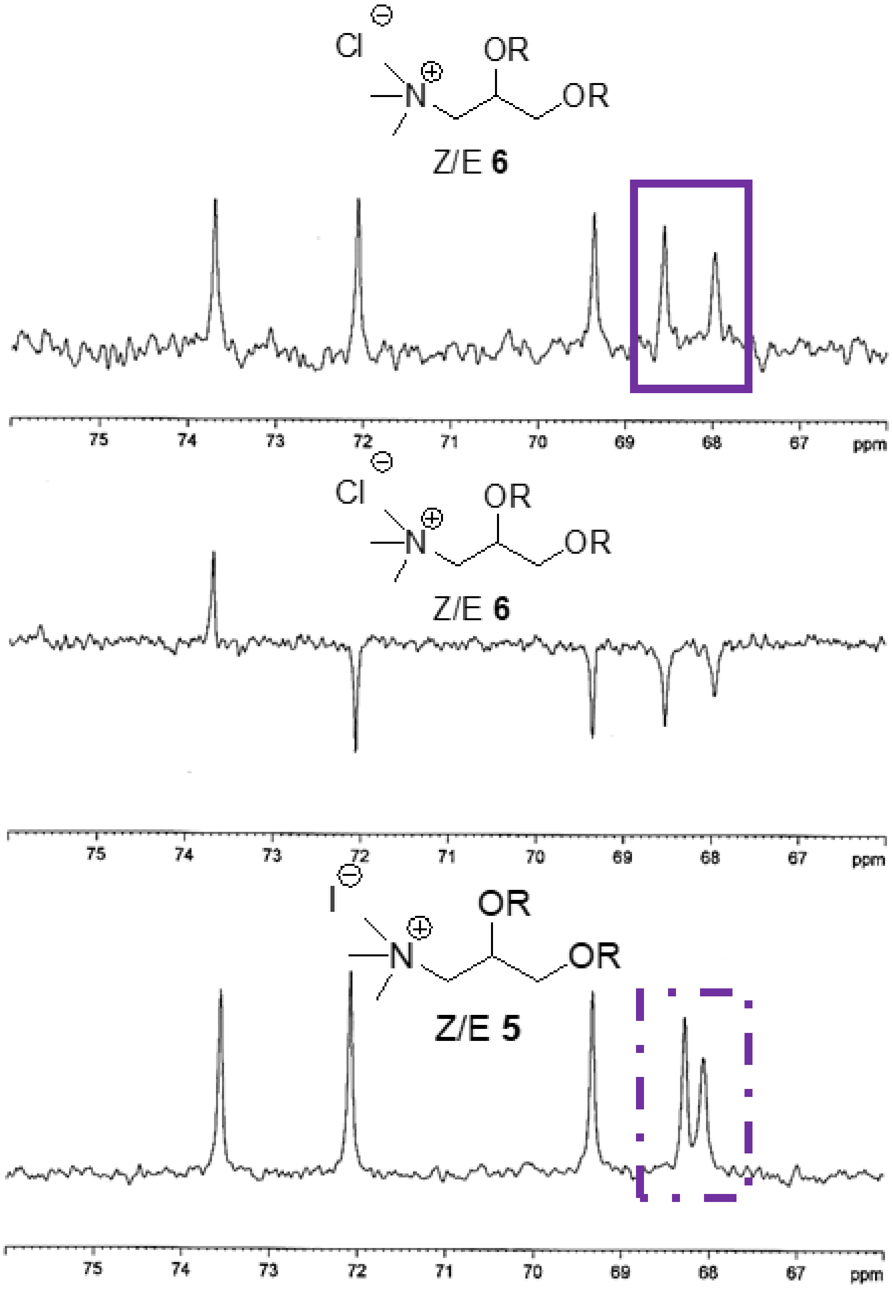

Figure S24. Comparison between the ^{13}C NMR spectra and DEPT-135 analysis ($CDCl_3$, 75.5 MHz) of Z/E **5** and Z/E **6**.

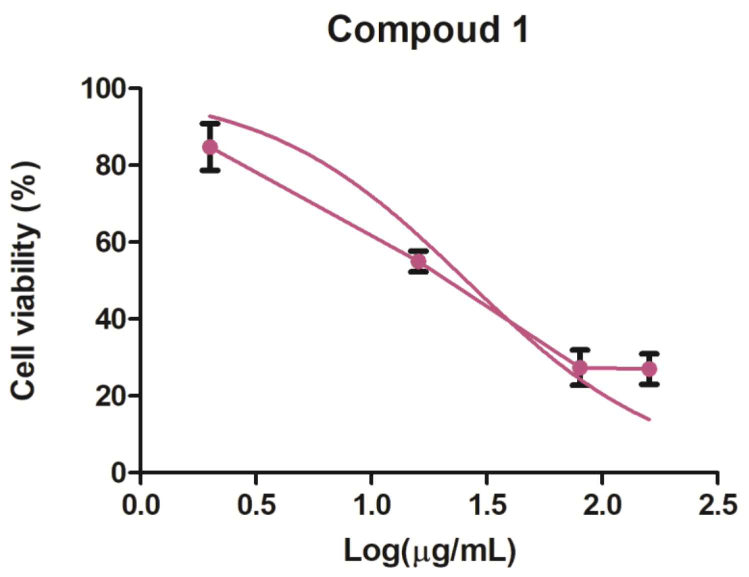

Figure S25. Plot of Log concentration of **1** vs. cell viability (%) (line with indicators and error bars) and plot of Log concentrations of **1** vs. normalized cell viability (%) (line without indicators).

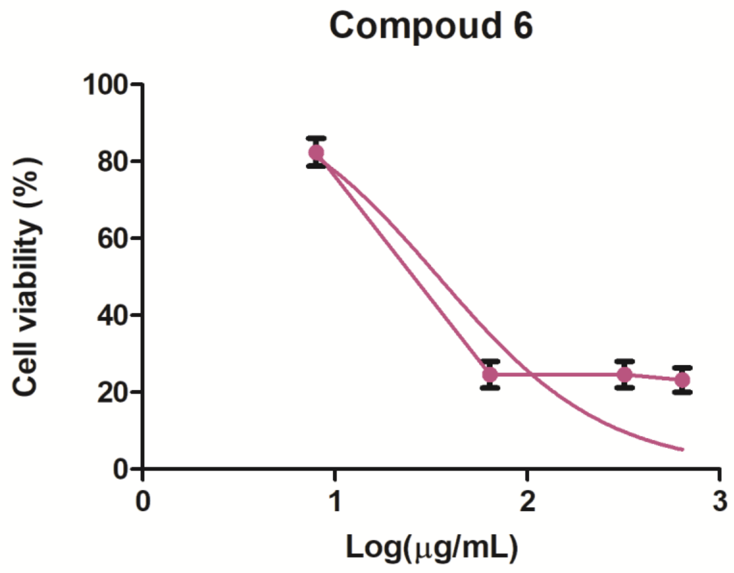

Figure S26. Plot of Log concentration of **6** vs. cell viability (%) (line with indicators and error bars) and plot of Log concentrations of **6** vs. normalized cell viability (%) (line without indicators).

Disclaimer/Publisher's Note: The statements, opinions and data contained in all publications are solely those of the individual author(s) and contributor(s) and not of MDPI and/or the editor(s). MDPI and/or the editor(s) disclaim responsibility for any injury to people or property resulting from any ideas, methods, instructions or products referred to in the content.
